# CoFe_2_O_4_-Graphene Nanocomposites Synthesized through An Ultrasonic Method with Enhanced Performances as Anode Materials for Li-ion Batteries

**DOI:** 10.1007/s40820-014-0003-7

**Published:** 2014-09-13

**Authors:** Yinglin Xiao, Xiaomin Li, Jiantao Zai, Kaixue Wang, Yong Gong, Bo Li, Qianyan Han, Xuefeng Qian

**Affiliations:** School of Chemistry and Chemical Engineering and State Key Laboratory of Metal Matrix Composites, Shanghai Jiao Tong University, Shanghai, 200240 People’s Republic of China

**Keywords:** Cobalt ferrite, Graphene, Anode materials, Lithium ion battery

## Abstract

CoFe_2_O_4_-graphene nanosheets (CoFe_2_O_4_-GNSs) were synthesized through an ultrasonic method, and their electrochemical performances as Li-ion battery electrode were improved by annealing processes. The nanocomposites obtained at 350 °C maintained a high specific capacity of 1,257 mAh g^−1^ even after 200 cycles at 0.1 A g^−1^. Furthermore, the obtained materials also have better rate capability, and it can be maintained to 696, 495, 308, and 254 mAh g^−1^ at 1, 2, 5, and 10 A g^−1^, respectively. The enhancements realized in the reversible capacity and cyclic stability can be attributed to the good improvement in the electrical conductivity achieved by annealing at appropriate temperature, and the electrochemical nature of CoFe_2_O_4_ and GNSs during discharge–charge processes.

CoFe_2_O_4_-graphene nanocomposites (CoFe_2_O_4_-GNSs) have been synthesized through an ultrasonic method combined with calcination process. The nanocomposite calcinated at 350 °C shows better rate capabilities, e.g., 696, 495, 308, and 254 mAh g^−1^ at 1, 2, 5, and 10 A g^−1^, respectively.

**Electronic supplementary material:**

The online version of this article (doi:10.1007/s40820-014-0003-7) contains supplementary material, which is available to authorized users.

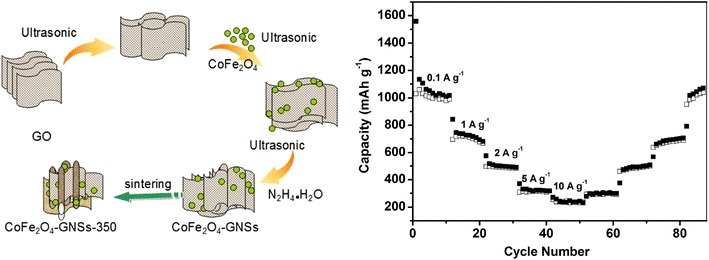

## Introduction

With the advantages of large specific capacity, high energy density, long cycle life, and environmental friendliness, lithium-ion batteries (LIBs) have become one of the predominant power sources for portable electronics in recent years [1–3]. However, the lower reversible storage capacity of carbon-based anode materials cannot match the demands for the practical applications in electric vehicles (EVs) and hybrid electric vehicles (HEVs) [4]. To meet the demands for these practical applications, the energy density, power density, cycle life, and rate performances of rechargeable LIBs need to be improved urgently [2, 5]. Transition-metal oxides (MO, where M is Co, Ni, Mn, Fe, Zn, or Cu) were found to be promising anode materials to replace carbonaceous anodes due to the former’s higher theoretical reversible capacities [2, 6] (e.g., 717 mAh g^−1^ of NiO [7], 1,007 mAh g^−1^ of Fe_2_O_3_ [8], 755 mAh g^−1^ of MnO [9], and 890 mAh g^−1^ of Co_2_O_3_ [10]). However, the huge volume changes during continuous charging/discharging processes would lead to the rapid disintegration of anodes caused by the induced mechanical stress and capacity fading upon cycling, which limited their further practical applications. To improve their electrochemical properties, especially for their cycling performances, and to overcome these problems, decreasing particle size into nanometer, doping metal into binary compounds (e.g., Al or Co), or fabricating nanocomposites has been used [11–14]. For example, Co_3_O_4_ nanorods/graphene nanocomposite had a discharge capacity of 1,310 mAh g^−1^ at a rate of 100 mA^−1^ after 40 cycles [15]; magnetite-modified graphene nanosheets (GNSs) exhibited remarkably high reversible lithium storage capacity (1,235 mAh g^−1^ at 0.2 A g^−1^ after 50 cycles), good rate capability (315 mAh g^−1^ at 10 A g^−1^), and improved cycling stability (450 mAh g^−1^ at 5 A g^−1^ after nearly 700 cycles) [3]. Recently, ternary oxide compounds, e.g., NiFe_2_O_4_, CuFe_2_O_4_, ZnFe_2_O_4_, ZnSnO_4_, ZnMn_2_O_4_, etc., have attracted considerable attention because of their good cyclic stability [16, 17]. For example, hollow CoFe_2_O_4_ nanosphere electrodes still retained more than 93.6 % reversible capacity of the first cycle even after 50 cycles. However, most of the ternary metal oxides suffer from the problem of poor electronic conductivity, and need to be modified by an electronically conductive agent, such as carbon nanotubes or graphene [18]. GNSs with superior properties, e.g., superior electrical conductivity, chemical inertness, and high surface area of over 2,600 m^2^ g^−1^ have attracted great interests in energy storage areas [19]. Thus, the electrochemical performances of GNSs/transition-metal oxide nanocomposites would be improved due to their synergistic effects by combining the high capacity of transition-metal oxides and high surface area/conductivity of GNSs, especially with regard to their rate capabilities and cycling performances [20, 21].

CoFe_2_O_4_, as a kind of important magnetic materials, has been the subject of intense research for the potential applications in high-density storage, magnetic resonance imaging, and drug-delivery technology [22]. Recently, the use of CoFe_2_O_4_ or its composites as anode materials for LIBs has become the hot topic of many researches due to its higher theoretical reversible capacities [23], and some methods have been developed [18, 24, 25]. For example, Xia and Liu et al. synthesized CoFe_2_O_4_-graphene nanocomposites by solvothermal method achieving the improvement of cycle performances, but the rate capability still needed to be further improved.

In this study, CoFe_2_O_4_-graphene nanocomposites have been prepared by sonication-assisted process as well as by annealing process at appropriate temperature. The sonication-assisted process can ensure the very good dispersion of CoFe_2_O_4_ nanoparticles in reduced graphene oxide [26]. The following appropriate annealing process can improve carbon quality, optimize the interface of graphene/nanoparticles, as well as improve the conductivity of graphene matrix [20, 27]. These combined effects make the obtained materials have better cyclic stability and rate capability compared with previous results.

## Experimental Section

### Synthesis of Graphite Oxide (GO), CoFe_2_O_4_, and CoFe_2_O_4_-GNSs Nanocomposites

GO was synthesized from natural graphite by the modified Hummer’s method [28]. CoFe_2_O_4_ was synthesized as follows: 1.4 mmol of CoCl_2_·6H_2_O, 2.52 mmol of FeCl_3_·6H_2_O, and 1.2 g of NaOH were mixed and dissolved in 40 mL distilled water after being sonicated for 30 min, and then transferred into a Teflon-line autoclave and maintained at 180 °C for 12 h. Final products were separated by centrifugation and dried in 80 °C.

CoFe_2_O_4_-GNSs nanocomposites were synthesized as follows: 0.2 g of GO was added into 50 mL water and sonicated for 30 min, and then 0.5 g of CoFe_2_O_4_ was added into the solution, followed by sonication for another 30 min. Finally, 10 mL of hydrazine hydrate was added into the mixtures followed by sonication for 60 min. The resulting products were centrifuged and washed by distilled water, and then dried in 80 °C. The obtained samples were further annealed at selected temperatures (e.g., 350, 550 °C, etc.) for 60 min at a heating rate of 10 °C min^−1^ in N_2_, which were named as CoFe_2_O_4_-GNSs-350 and CoFe_2_O_4_-GNSs-550 corresponding to their annealing temperature.

### Characterizations

The obtained products were characterized by XRD (Shimadzu XRD-6000, CuKα, 40 kV, 30 mA). Raman spectra were recorded on a Super LabRam-II spectrometer with a holographic grating of 1,800 g mm^−1^. Morphology of samples was investigated using a transmission electron microscopy (TEM) system (JEOL, JEM-2100). Thermogravimetric (TG) analysis was carried out under air on a Perkin-Elmer 7 instrument to determine the weight ratio of GNSs to CoFe_2_O_4_. Nitrogen adsorption–desorption measurement was conducted at 77.7 K on a Micromeritics ASAP 2010 analyzer.

### Elctrochemical Testing

Working electrodes were prepared by mixing a slurry containing 80 % active material (CoFe_2_O_4_-GNSs nanocomposites), 10 % acetylene black, and 10 % polymer binder (polyvinylidene difluoride, PVDF) on copper foil according to previous works [29, 30], and then dried in a vacuum oven at 60 °C for 12 h. Electrolyte consisted of a solution of 1 mol L^−1^ of LiClO_4_ in ethylene carbonate (EC)/diethylene carbonate (DEC) (1:1 vol %). Charge–discharge cycles of cells were evaluated between 0.01 and 3 V at a current density of 100 mA g^−1^ for the first cycle using a battery test system (LAND CT2001A model, Wuhan Jinnuo Electronics, Ltd.). All tests were performed at room temperature. Electrochemical impedance spectroscopy (EIS) was performed using an Ametek PARSTAT 2273 electrochemistry workstation.

## Results and Discussion

**Fig. 1:**
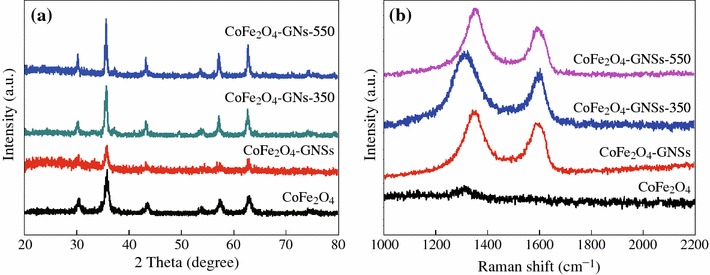
XRD patterns (**a**) and Raman spectra (**b**) of CoFe_2_O_4_, CoFe_2_O_4_-GNSs, CoFe_2_O_4_-GNSs-350, and CoFe_2_O_4_-GNSs-550, respectively

XRD patterns of the obtained CoFe_2_O_4_, CoFe_2_O_4_-GNSs, CoFe_2_O_4_-GNSs-350, and CoFe_2_O_4_-GNSs-550 nanocomposites are shown in Fig. 1a; all main diffraction peaks can be readily indexed to rhombohedral CoFe_2_O_4_ (JCPDS card No. 79-1,744, space group: R-3m, *a* = 5.94 Å). In the Raman spectra of CoFe_2_O_4_-GNSs, CoFe_2_O_4_-GNSs-350, and CoFe_2_O_4_-GNSs-550 nanocomposites, the two weak peaks at 1,325 and 1,600 cm^−1^ are respectively assigned to the D band and G band of graphene [30], and their relative intensity (*I*_D_/*I*_G_ ratio) relates the quantity of restoration of the sp^2^ carbon [31, 32], and the restoration of the sp^2^ carbon results in the increasing of conductivity of GNSs [33, 34]. From Fig. 1b, one can see that the *I*_D_/*I*_G_ ratio increases with the increase of annealing temperature because of the removal of oxygen-containing groups [33, 34]. Interestingly, the D peaks of GNSs in CoFe_2_O_4_-GNSs-350 nanocomposites show the obvious red-shift, revealing the stronger interactions between CoFe_2_O_4_ nanoparticles and GNSs after being annealed at 350 °C. This phenomenon usually derives from the dielectric confinement effect of transition metal oxide on GNSs. However, if the annealing temperature is increased to 550 °C, the interactions would become weaker due to the removal of functional group and the enlarged particle size of CoFe_2_O_4_ nanoparticles. As a result, the position of *D* band of CoFe_2_O_4_-GNSs-550 shows similar position to that of CoFe_2_O_4_-GNSs.

**Fig. 2:**
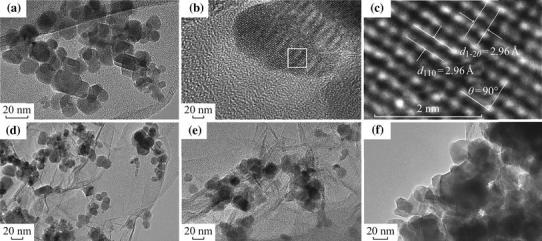
TEM images of bare CoFe_2_O_4_ (**a**); CoFe_2_O_4_-GNSs (**d**); CoFe_2_O_4_-GNSs-350 (**e**); and CoFe_2_O_4_-GNSs-550 (**f**). HRTEM images of CoFe_2_O_4_ (**b**, **c**)

**Fig. 3:**
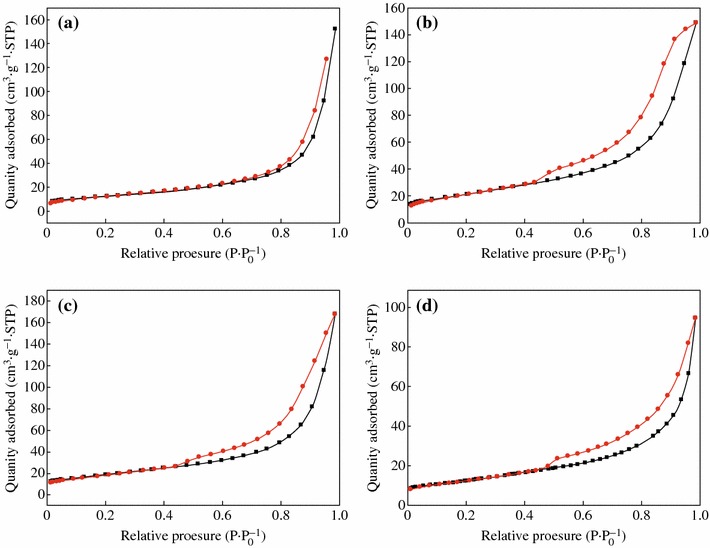
Nitrogen adsorption/desorption isotherms of CoFe_2_O_4_ (**a**), CoFe_2_O_4_-GNSs (**b**), CoFe_2_O_4_-GNSs-350 (**c**), and CoFe_2_O_4_-GNSs-550 (**d**)

Figure 2 shows the TEM images of CoFe_2_O_4_, CoFe_2_O_4_-GNSs, CoFe_2_O_4_-GNSs-350, and CoFe_2_O_4_-GNSs-550 nanocomposites, and HRTEM images of CoFe_2_O_4_ nanoparticles. From Fig. 2a, one can see that CoFe_2_O_4_ nanoparticles with the size about 20 nm are the main products. The 2.96 Å interplanar distance corresponds to the (1 –2 0) crystal plane and the 2.96 Å lattice spacing corresponds to the (1 1 0) planes of rhombohedral CoFe_2_O_4_ in Fig. 2b–c; the angle of 90° between (1 1 0) and (1 –2 0) planes matches well with its crystal structure (shown in Fig. S1), implying that the obtained CoFe_2_O_4_ nanoparticles have good crystallinity as well. As shown in the TEM images of CoFe_2_O_4_-GNSs nanocomposites (Fig. 2d), CoFe_2_O_4_ nanoparticles disperse homogenously on the paper-like GNSs, which can prevent the stack of GNSs layers and form a 3D laminated structure. From the TEM images of CoFe_2_O_4_-GNSs-350 nanocomposites (Fig. 2e), a closer contact of CoFe_2_O_4_ nanoparticles with GNSs can be observed, which is beneficial for increasing the conductivity of electrodes, ensures the fast and sustained transportation of electrons in electrodes, and enhances Li-ion diffusion rate during electrochemical reaction. However, more coalescence would happen along the grain boundaries of CoFe_2_O_4_ nanoparticles with the increasing calcination temperature (Fig. 2f) [35], which would lead to lower capacities because of longer diffusion length of Li-ion and poor conductivity of electrode. Nitrogen adsorption/disadsorption isotherms of CoFe_2_O_4_, CoFe_2_O_4_-GNSs, CoFe_2_O_4_-GNSs-350, and CoFe_2_O_4_-GNSs-550 (Fig. 3) reveal their specific surface areas as 24.04, 137.13, 106.23, and 55.64 m^2^ g^−1^, respectively. The larger surface area of CoFe_2_O_4_-GNSs nanocomposites over CoFe_2_O_4_ is derived from GNSs. Compared with CoFe_2_O_4_-GNSs nanocomposites, the annealing process would lead to smaller specific surface area because of the shrinkage of GNSs and/or agglomeration of CoFe_2_O_4_ nanoparticles, and CoFe_2_O_4_-GNSs-550 nanocomposite would have the lowest specific surface area [33, 36, 37].

**Fig. 4:**
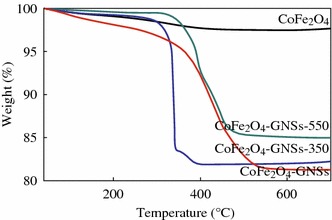
Thermogravimetry analyses (TGA) of CoFe_2_O_4_, CoFe_2_O_4_-GNSs, CoFe_2_O_4_-GNSs-350, and CoFe_2_O_4_-GNSs-550

The weight ratios of GNSs in CoFe_2_O_4_-GNSs, CoFe_2_O_4_-GNSs-350, and CoFe_2_O_4_-GNSs-550 nanocomposites were evaluated by thermal gravimetric analysis (TGA) under air flow (Fig. 4). The final residues are CoFe_2_O_4_ [35, 37], and the weight loss of CoFe_2_O_4_-GNSs may correspond to the oxidation of GNSs to CO_2_. According the changed weights of CoFe_2_O_4_, CoFe_2_O_4_-GNSs, CoFe_2_O_4_-GNSs-350, and CoFe_2_O_4_-GNSs-550 nanocomposites, the weight percentages of GNSs in CoFe_2_O_4_-GNSs, CoFe_2_O_4_-GNSs-350, and CoFe_2_O_4_-GNSs-550 nanocomposites are about 18, 17.5, and 15 %, respectively.

**Fig. 5:**
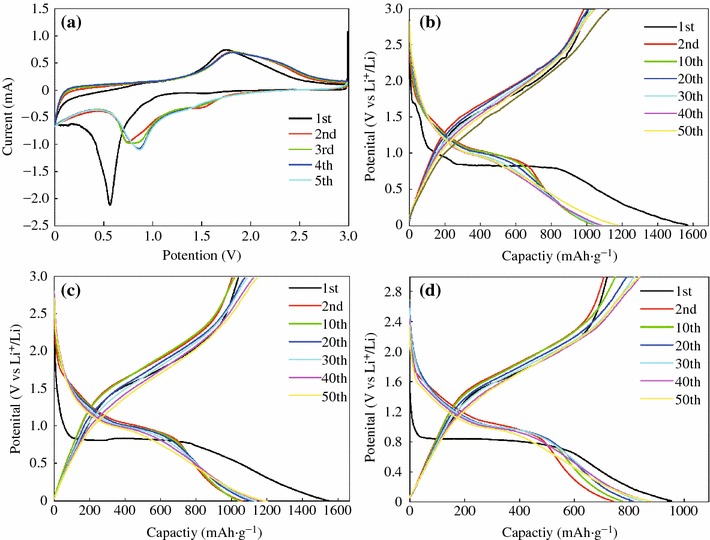
Cyclic voltammograms of CoFe_2_O_4_-GNSs nanocomposites (**a**) for the first five cycles between 3.00 and 0.01 V versus Li. Charge–discharge curves at 0.1 A g^−1^: CoFe_2_O_4_-GNSs (**b**), CoFe_2_O_4_-GNSs-350 (**c**); and CoFe_2_O_4_-GNSs-550 (**d**)

The electrochemical performances of the as-prepared CoFe_2_O_4_-GNSs nanocomposites as anode materials for LIBs were studied, and the cyclic voltammograms (CVs) between 0 and 3.0 V at a scan rate of 0.005 V s^−1^ are shown in Fig. 5a. In the first cycle, a smaller cathodic peak below 0.25 V (vs. Li/Li^+^) is associated with the reactions of Li intercalation into GNSs and the porous structure of CoFe_2_O_4_-GNSs nanocomposites. The large cathodic peak at 0.60 V (vs. Li/Li^+^) is associated with the reduction reactions of CoFe_2_O_4_ by Li during the first discharge process, which is similar to the previous reports [1]. This process can be expressed by following reaction:

1 $${\text{CoFe}}_{2}{\text{O}}_{4}+8{\text{Li}}^{+}+8\text{e}\to \text{Co}+2\text{Fe}+4{\text{Li}}_{2}\text{O}$$

The anodic peak located at 1.70 V may be corresponding to the oxidation of metallic iron and cobalt, and it shifts positively to 1.75 V in the second and subsequent cycles; while the corresponding cathodic peak shifts to 0.7 V in the second cycles and then shifts to 0.85 V in the third cycles and subsequent cycles because of the polarization of electrode materials [38]. This process can be expressed by following reactions:

2 $$\text{Co}+{\text{Li}}_{2}\text{O}\to \text{CoO}+2{\text{Li}}^{+}+2\text{e}$$

3 $$2\text{Fe}+3{\text{Li}}_{2}\text{O}\to {\text{Fe}}_{2}{\text{O}}_{3}+6{\text{Li}}^{+}+6\text{e}$$

In the second cycle, the peak at 1.5 V in the cathodic process can be attributed to the Faradic capacitance both on the surface and edge sites of GNSs [39, 40]. After the third cycle, reversible cathodic and anodic peaks are located at around 0.85 and 1.75 V because of the reversible oxidation/reduction processes during charge/discharge cycles, respectively. These processes can be expressed by the following reaction:

4 $$\text{Co}+2\text{Fe}+4{\text{Li}}_{2}\text{O}↔\text{CoO}+{\text{Fe}}_{2}{\text{O}}_{3}+8\text{Li}$$

Furthermore, the CV curves of CoFe_2_O_4_-GNSs electrode are stable and well overlapped, and the integral area and peak intensity in the fifth cycle are close to that of the fourth cycle, implying that the electrochemical reversibility of the obtained nanocomposites is gradually built after the third cycle and possesses good capacity retention [41].

**Fig. 6:**
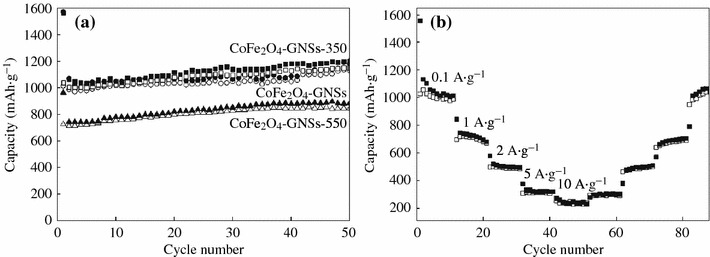
Electrochemical performances of CoFe_2_O_4_-GNSs nanocomposites: Circle stability at 0.1 A g^−1^ for CoFe_2_O_4_-GNSs, CoFe_2_O_4_-GNSs-350, and CoFe_2_O_4_-GNSs-550 (**a**); Rate capability of CoFe_2_O_4_-GNSs-350 (**b**)

The discharge–charge voltage profiles of CoFe_2_O_4_-GNSs, CoFe_2_O_4_-GNSs-350, and CoFe_2_O_4_-GNSs-550 nanocomposites at a current density of 0.1 A g^−1^ are shown in Fig. 5b–d. The platform at 0.85 V is associated with the reduction reaction of CoFe_2_O_4_ by Li during the first discharge process. The electrodes based on CoFe_2_O_4_-GNSs, CoFe_2_O_4_-GNSs-350, and CoFe_2_O_4_-GNSs-550 nanocomposites with coulombic efficiencies of about 64, 66, and 75 %, respectively, deliver discharge capacities of 1,509, 1,508, and 959 mAh g^−1^ in the first cycle, respectively. The capacities are much larger than the theoretical value of CoFe_2_O_4_ (912 mAh g^−1^), and the extra irreversible capacities can be attributed to the solid electrolyte interphase (SEI) films [42]. However, they have reversible discharge capacities of 973, 986, and 721 mAh g^−1^ in the second cycle, and the larger reversible capacities can be attributed to the decomposition of organic electrolytes and the amorphism of CoFe_2_O_4_ nanoparticles. Similar phenomena also have been observed in other transition metal oxides [43]. Moreover, the efficiencies of CoFe_2_O_4_-GNSs, CoFe_2_O_4_-GNSs-350, and CoFe_2_O_4_-GNSs-550 rapidly increase to 95, 95, and 96 % in the third cycle, respectively, and remain thus in the following cycles. Furthermore, the reversible capacities of CoFe_2_O_4_-GNSs, CoFe_2_O_4_-GNSs-350, and CoFe_2_O_4_-GNSs-550 nanocomposites slightly increase from the second cycle and reach to ~1,086, 1,071, and 839 mAh g^−1^ after 50 cycles, respectively, which could be ascribed to the gradual activation of GNSs in nanocomposites in the first several cycles. On the other hand, porous structure can form during discharge–charge processes (Fig. S2), and these in situ-formed porous structures also have a contribution to their reversible capacities [44, 45]. Figure 6a shows the discharge–charge cycling performances of CoFe_2_O_4_-GNSs, CoFe_2_O_4_-GNSs-350, and CoFe_2_O_4_-GNSs-550 nanocomposites at a current density of 0.1 A g^−1^. From Fig. 6a, one can see that the capacities of CoFe_2_O_4_-GNSs, CoFe_2_O_4_-GNSs-350, and CoFe_2_O_4_-GNSs-550 nanocomposites at a current density of 0.1 A g^−1^ are about 973, 986, and 721 mAh g^−1^ in the second cycle, respectively, and they still have capacities of 1,086, 1,071, and 839 mAh g^−1^ after 50 cycles, which are about 111, 108, and 118 % of the reversible capacity of the first cycle. The gradual increase in capacity after the 50th cycle is attributed to the reversible polymerization/oligomerization of carbonates and alkyl carbonates (main components of electrolyte), which would further lead to form a reversible polymeric/gel films on nanocomposites [21, 46]. Moreover, the capacity of CoFe_2_O_4_-GNSs-350 nanocomposites still keeps increasing to 1,257 mAh g^−1^ after 200 cycles at the current of 0.1 A g^−1^ (Fig. S3a). The long-term cyclic stabilities of all the nanocomposites can be due to the electrochemical nature of multiple metal oxide and very good dispersion of CoFe_2_O_4_ nanoparticles into graphene matrix created by the sonication-assisted process.

The capacities of CoFe_2_O_4_-GNSs, CoFe_2_O_4_-GNSs-350, and CoFe_2_O_4_-GNSs-550 nanocomposites at a current density of 1 A g^−1^ are 776, 775, and 525 mAh g^−1^ in the second cycle, and change to 436, 596, and 298 mAh g^−1^ after 180 cycles (Fig. S3b), respectively. However, the capacity of CoFe_2_O_4_ nanoparticles only changes to 11 mA h g^−1^ after 180 cycles at the same conditions, which is only about 2 % capacity of the first cycle. Figure 6b illustrates the rate capability of CoFe_2_O_4_-GNSs-350 nanocomposites at the current densities ranging from 0.1 to 10 A g^−1^. It can be seen that the reversible capacities change to 1,029, 696, 495, and 308 mAh g^−1^ at 0.1, 1, 2, and 5 A g^−1^, respectively; however, CoFe_2_O_4_-GNSs-350 electrode can still keep a stable reversible capacity of 254 mAh g^−1^ even at the current density as high as 10 A g^−1^. Moreover, the capacities still change to 294, 461, 638, and 1,016 mAh g^−1^ when the current densities return to 5, 2, 1, and 0.1 A g^−1^, respectively, indicating that the obtained CoFe_2_O_4_-GNSs-350 nanocomposites exhibit remarkable high lithium storage capacity with the improved reversible cycling stability and superior rate capability. The better rate performances of the obtained CoFe_2_O_4_-graphene nanocomposites, compared with that of Xia (372 mAh g^−1^ at 2 A g^−1^) and that of Liu (440 mAh g^−1^ at 1.6 A g^−1^), may be due to the improved electrochemical performances, created by the properly annealed process.

**Fig. 7:**
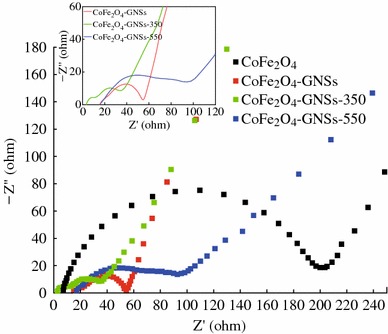
Nyquist plots of CoFe_2_O_4_, CoFe_2_O_4_-GNSs, CoFe_2_O_4_-GNSs-350, and CoFe_2_O_4_-GNSs-550 at 0.08 V versus Li after 5 cycles

To further investigate the effects of GNSs in CoFe_2_O_4_-GNSs nanocomposites, Nyquist plots of CoFe_2_O_4_, CoFe_2_O_4_-GNSs, CoFe_2_O_4_-GNSs-350, and CoFe_2_O_4_-GNSs-550 nanocomposites after five cycles are shown in Fig. 7. The Nyquist plot of CoFe_2_O_4_ shows a typical semicircle derived from the charge transfer impedance through the electrode/electrolyte interface, while the Nyquist plots of all nanocomposites have multiple semicircles because of the charge-transfer impedance through the interface of electrode/electrolyte and the inside charge-transfer impedances [47–49]. The electrode based on CoFe_2_O_4_-GNSs-350 nanocomposite has the smallest multiple semicircles, indicating its lowest charge-transfer impedance. Compared with the works of Xia and Liu [24, 25], appropriate annealing process can improve the conductivity of the obtained CoFe_2_O_4_-GNSs nanocomposites because it can optimize the interfaces of graphene/nanoparticles and strengthen the interactions between CoFe_2_O_4_ and GNSs [20, 27]. However, high annealing temperature (550 °C) would lead to the serious agglomeration of nanoparticles and further increase the resistance.

Based on above discussion, the improvement of electrochemical performances of CoFe_2_O_4_-GNSs nanocomposites can be attributed to their unique structure and electrochemical nature. First, CoFe_2_O_4_ can transform into nanosized hybrid of Fe_2_O_3_/CoO during the first discharge process [1]. The in situ-formed hybrid of Fe_2_O_3_/CoO can combine with GNSs to form a porous structure, which can further accommodate its volume change and result in good stability of electrode. Similar phenomena have also been observed in MnFe_2_O_4_-GNSs nanocomposites [50]. On the other hand, the in situ-formed nanosized Fe_2_O_3_ (CoO) can also act as the matrix of CoO (Fe_2_O_3_), which would prevent the aggregations of Fe_2_O_3_ (CoO), and accommodate the volume change of active materials during discharge–charge processes and further improve their cycle stability. The obtained porous structure can accommodate its volume change and result in good stability of electrode [51]. Second, GNSs fabricated from the annealing process can increase the conductivity of electrodes, ensure the fast and sustained transportation of electrons in electrodes, and enhance Li-ion diffusion rate during electrochemical reaction. Third, the annealing process can increase the interfaces of graphene/grain and grain/grain, and facilitate the ion/charge transfer during charge/discharge, which can be confirmed by EIS and TEM images. Furthermore, GNSs may also increase the BET surface area of nanocomposites and provide extra space for buffering the volumetric change, which would avoid the cracking of electrodes and maintain the structural integrity of electrodes during continuous charging/discharging. Finally, GNSs can also prevent the agglomeration CoFe_2_O_4_ nanoparticles because no larger nanoparticles are observed in Fig. 3d, which is also beneficial for improving the electrochemical performance of CoFe_2_O_4_-GNSs nanocomposites.

## Conclusion

CoFe_2_O_4_-GNSs, CoFe_2_O_4_-GNSs-350, and CoFe_2_O_4_-GNSs-550 nanocomposites have been synthesized through an ultrasonic and annealing process, and they exhibit different electrochemical performances of lithium storage capacity with improved reversible cycling stability and superior rate capability, e.g., the capacities of CoFe_2_O_4_-GNSs, CoFe_2_O_4_-GNSs-350 and CoFe_2_O_4_-GNSs-550 nanocomposites are about 1,086, 1,071, and 839 mAh g^−1^ in the second cycles, and they still increase to 1,128, 1,148, and 839 mAh g^−1^ after 50 cycles, respectively. Moreover, CoFe_2_O_4_-GNSs-350 nanocomposites have the reversible capacities of about 1,257 and 596 mAh g^−1^ even after 200/180 cycles at current densities of 0.1 and 1 A g^−1^, respectively. Furthermore, the obtained CoFe_2_O_4_-GNSs-350 nanocomposites also have better rate capability, and it can be maintained to 308 and 254 mAh g^−1^ at 5 and 10 A g^−1^, respectively. The long-term cyclic stability can be due to the electrochemical nature and better dispersion of CoFe_2_O_4_ nanoparticles in graphene matrix created by the sonication-assisted process. Furthermore, proper annealing process can improve graphene quality and optimize the interfaces of graphene/grain and grain/grain and further improve the conductivity of obtained materials, which would further lead to the improved rate capability.

## Electronic supplementary material

Below is the link to the electronic supplementary material.

Supplementary material 1 (DOC 1896 kb)
