# Supplementary material for: CoFe2O4-Graphene Nanocomposites Synthesized through An Ultrasonic Method with Enhanced Performances as Anode Materials for Li-ion Batteries
Source: Nanomicro Lett. 2014 Sep 13;6:307–15. doi: 10.1007/s40820-014-0003-7 (PMC6223971; doi:10.1007/s40820-014-0003-7)
Supplement: Supplementary file 1 — Supplementary material 1 (DOC 1896 kb) [file 40820_2014_3_MOESM1_ESM.doc]

***Highlights:***

CoFe_2_O_4_-graphene nanocomposites were prepared by sonication-assisted process combined with calcination.

The obtained materials show high reversible capacities of (1257 mAh g^-1^ at 0.1 A g^-1^) and improved rate capability (596 mAh g^-1^ at1 A g^-1^).

The improvement can be attributed to well disperse CoFe_2_O_4_ and enhanced conductivity derived from the combined fabrication process.

***Supporting Information for***

**CoFe_2_O_4_-graphene Nanocomposites Synthesized through An Ultrasonic Method with Enhanced Performances as Anode Materials for Li-ion Batteries**

Yinglin Xiao, Xiaomin Li, Jiantao Zai*, Kaixue Wang, Yong Gong, Bo Li, Qianyan Han and Xuefeng Qian*

School of Chemistry and Chemical Engineering and State Key Laboratory of Metal Matrix Composites, Shanghai Jiao Tong University, Shanghai, 200240, P.R. China

*Corresponding authors. E-mail: [xfqian@sjtu.edu.cn](mailto:xfqian@sjtu.edu.cn)

**Figure S1** The [crystal](app:ds:crystal) [structure](app:ds:structure) of CoFe_2_O_4_.

**Figure S2** HRTEM image of CoFe_2_O_4_-GNSs-350 based electrode materials after 50 discharge-charge processes.

**Figure S3** Circle stability at 0.1 A g^-1^ for CoFe_2_O_4_-GNSs-350 (a); and 1 A g^-1^ for CoFe_2_O_4_, CoFe_2_O_4_-GNSs, CoFe_2_O_4_-GNSs-350 and CoFe_2_O_4_-GNSs-550 (b).


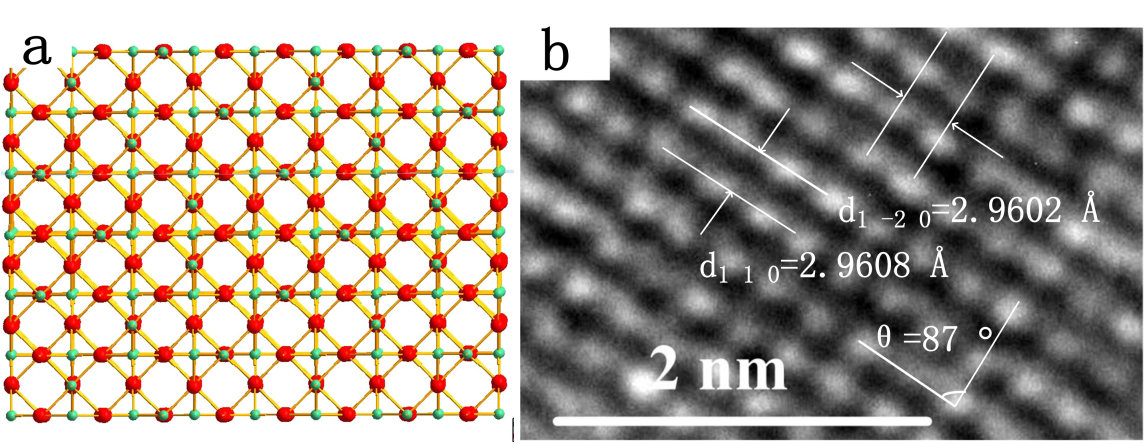


Fig. S1


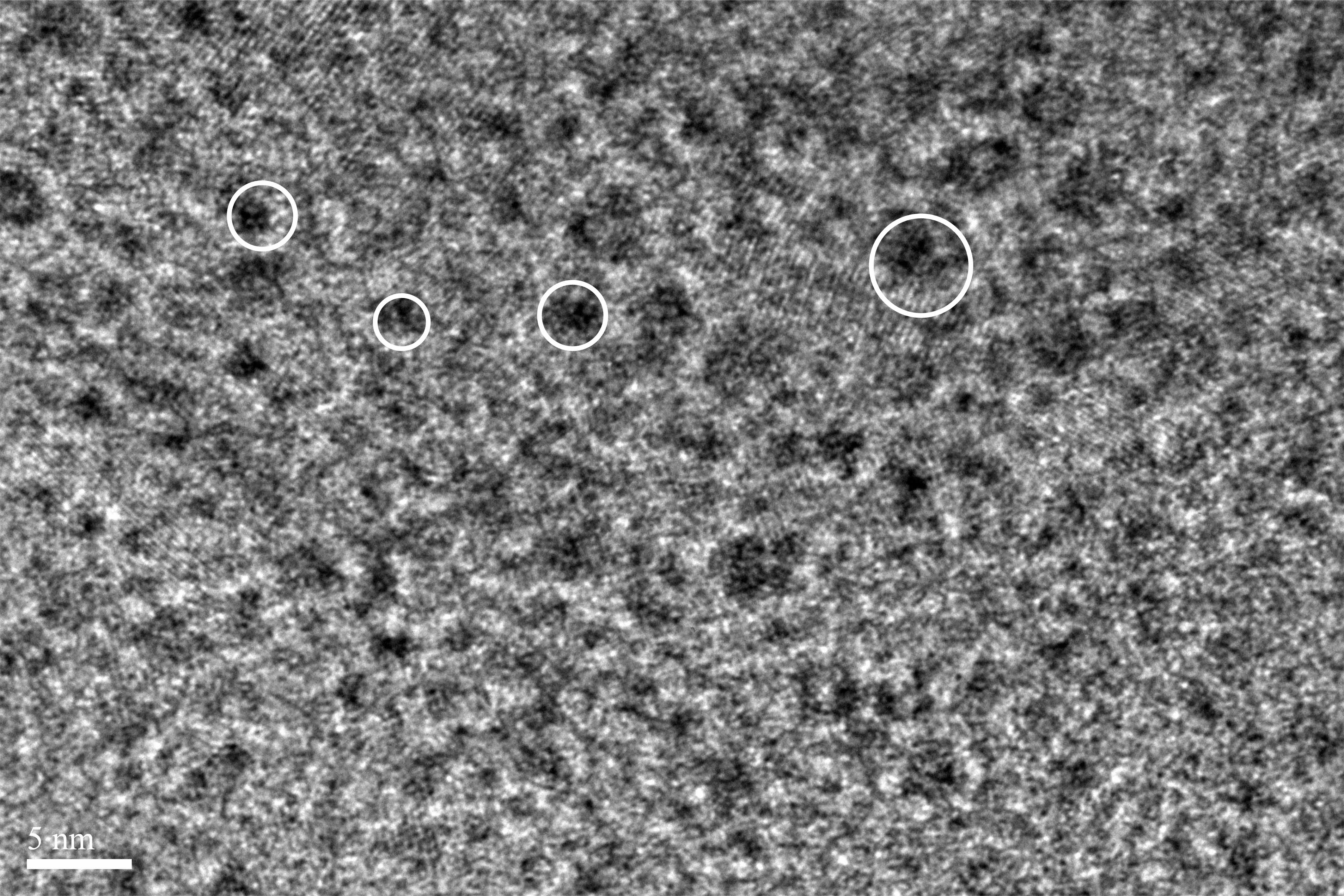


Fig. S2


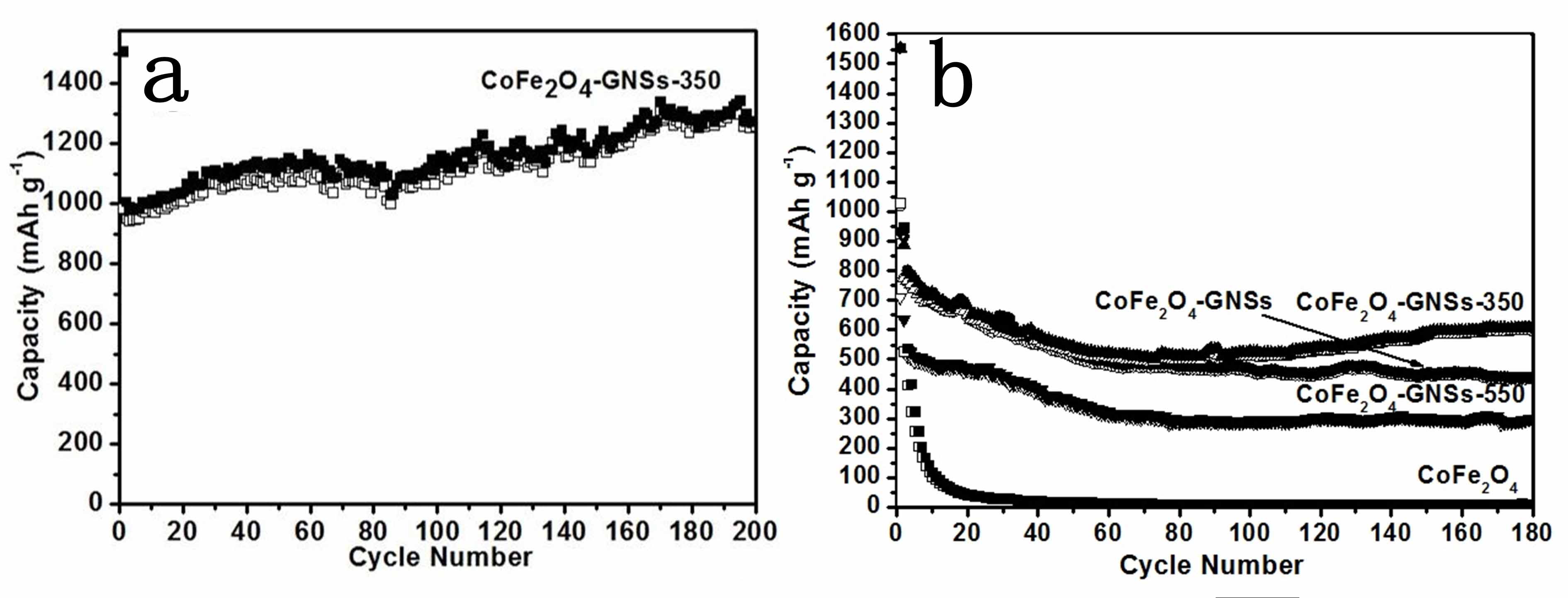


Fig. S3
